# Supplementary material for: Identification of photoperiod-induced specific miRNAs in the adrenal glands of Sunite sheep (Ovis aries)
Source: Front Vet Sci. 2022 Jul 22;9:888207. doi: 10.3389/fvets.2022.888207 (PMC9354845; doi:10.3389/fvets.2022.888207)
Supplement: Supplementary file 1 [file Data_Sheet_1.ZIP › Supplementary file/miRDeep2 file.zip!/miRDeep2 file/Know_Structure/SP42A1/oar-mir-431.pdf]

miRBase precursor : oar-mir-431
Total read count : 111
oar-miR-431 read count : 10
remaining reads : 101

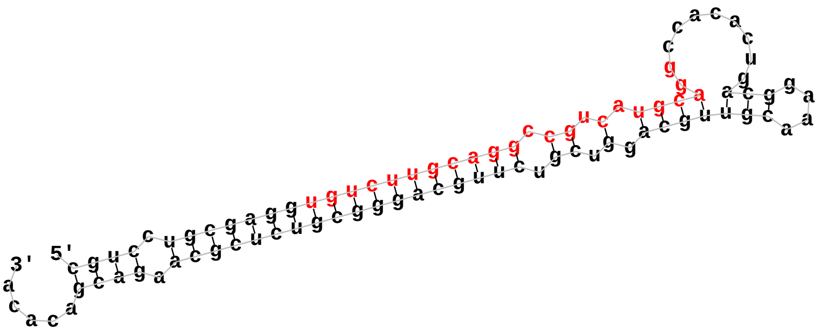


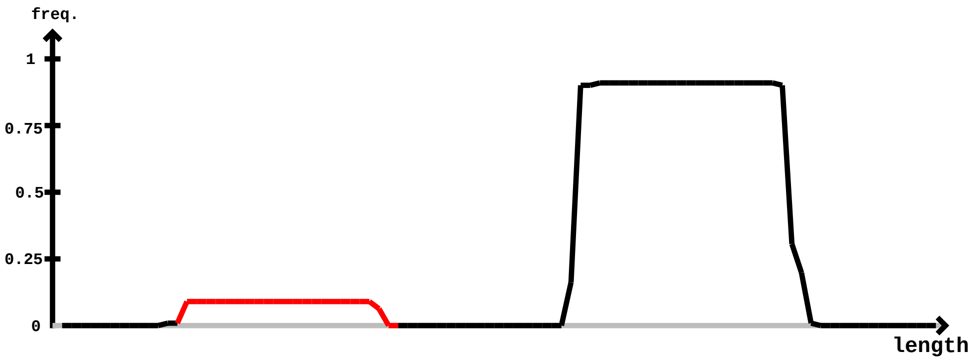


```
                oar-miR-431
5'- cguccugcgaggugucuugcaggccgucaugcaggccacacugacggaaacguugcaggucgucuugcagggcgucucgcaagacgacaca   -3'    exp
    ((((.((((((((((((((((((.((.(.((((..........(((....))))))).).)).)))))))))))))))))).)))).....    reads      mm         sample
    ..........ggugucuugcaggccgucaugc...........................................................    1          0          seq
    ............ugucuugcaggccgucaugc...........................................................    2          0          seq
    ............ugucuugcaggccgucaugca..........................................................    7          0          seq
    ....................................................uugcaggucgucuugcagggcgu................    14         0          seq
    ....................................................uugcaggucgucuugcagggcguc...............    2          0          seq
    ....................................................uugcaggucgucuugcagggcgucu..............    2          0          seq
    .....................................................ugcaggucgucuugcagggcg.................    1          0          seq
    .....................................................ugcaggucgucuugcagggcgu................    52         0          seq
    .....................................................ugcaggucgucuugcagggcguc...............    9          0          seq
    .....................................................ugcaggucgucuugcagggcgucu..............    19         0          seq
    .....................................................ugcaggucgucuugcagggcgucuc.............    1          0          seq
    .......................................................caggucgucuugcagggcguc...............    1          0          seq
```
